# Evolutionary Influences on Proteins

**DOI:** 10.1371/journal.pbio.0050026

**Published:** 2007-02-06

**Authors:** Rachel Jones

The strings of amino acids that make up a protein are specified by the sequence of nucleotides in the coding region of a gene. However, genes also contain nucleotides that don't contribute to the sequence of a protein, in noncoding areas called introns. Before the protein can be generated, the introns must be removed and the coding parts (called exons) must be spliced back together.

Splice-enhancer domains—exon sequences near the intron–exon boundary—help to ensure that genes are spliced at the correct points. They also code for specific amino acids within the protein, indicating that they must serve two functions. In a new study, Joanna Parmley, Laurence Hurst, and colleagues asked what effect this dual functionality has on the evolution of these sequences. They found evidence that the necessity for splice enhancers near the intron–exon boundaries causes these sequence regions to evolve at a lower-than-average rate. The region near to intron–exon boundaries is also more likely than expected to contain nucleotide sequences that are used in splice-enhancer regions—a condition that results in a skewed amino acid content in the corresponding parts of the encoded proteins. Thus, the amino acid sequence of a protein might depend not only on its biological function, but also on the presence of splice enhancers.

The authors began by analyzing the use of different amino acids near intron–exon boundaries. Most amino acids were either more or less abundant than expected by chance in these regions. The more-abundant ones were encoded by nucleotide sequences found in splice enhancers. When the authors analyzed amino acids that can be encoded by several different triplets of nucleotides (each amino acid is encoded by three nucleotides), they found that the increased abundance of the amino acids probably resulted from a preference for specific nucleotides, rather than a direct preference for those amino acids.

**Figure:**
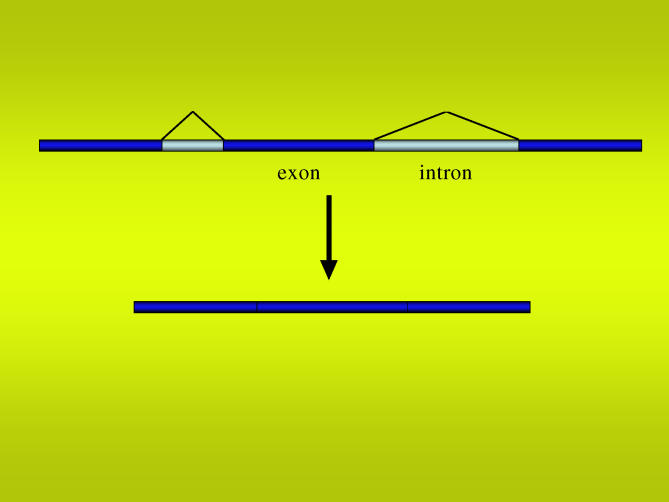
Before a transcribed gene is translated into the amino acids of its encoded protein, noncoding intron sequences are removed and the remaining coding exons are spliced together.

If there is selection pressure to conserve splice enhancers—that is, if the splice enhancers confer some sort of evolutionary or fitness benefit and thus are preserved by natural selection—one would expect these regions to evolve more slowly than other parts of the genetic sequence. By comparing splice-enhancer sequences in mouse and human genes, the authors showed that the sequences are in fact conserved—and that smaller exons, in which more of the nucleotides are close to an intron–exon boundary, also evolve more slowly.

The rate of evolution of a protein is also constrained by other factors. For example, “housekeeping” genes—those whose proteins are essential for cellular function and are expressed in many tissues—tend to evolve slowly, whereas nonessential genes (whose functions might be reproduced by another, similar genes) often evolve more quickly. The results of this study show that the proportion of a gene that falls near intron–exon boundaries has a strong effect on the rate of protein evolution when compared with these other factors.

An interesting insight into the possible functional effects of splice enhancers comes from looking at genes that have lost their introns. Such genes show markedly accelerated evolution in the regions that originally flanked intron–exon boundaries. This indicates that a selection constraint—presumably to maintain correct splicing by conserving splicing-enhancer domains—has been released following the loss of introns (because the splice enhancers are no longer needed). is finding also implies that the need to conserve the splice enhancers in the original proteins meant that the proteins were not optimized for their biological functions, but rather might have evolved a “compromise” sequence that could fulfil both roles.

The idea that the evolution of a gene can be so strongly influenced by something other than the biology of the protein it encodes is an intriguing one that might have consequences for gene therapy and for protein engineering, as well as for our understanding of protein evolution. Further work will be required to investigate whether other features, apart from splice-enhancer regions, also influence nucleotide and amino acid use near the boundaries between coding and noncoding gene segments.

